# How far is brain-inspired artificial intelligence away from brain?

**DOI:** 10.3389/fnins.2022.1096737

**Published:** 2022-12-09

**Authors:** Yucan Chen, Zhengde Wei, Huixing Gou, Haiyi Liu, Li Gao, Xiaosong He, Xiaochu Zhang

**Affiliations:** ^1^Hefei National Research Center for Physical Sciences at the Microscale, and Department of Radiology, the First Affiliated Hospital of USTC, Division of Life Science and Medicine, University of Science & Technology of China, Hefei, China; ^2^Department of Psychology, School of Humanities and Social Sciences, University of Science and Technology of China, Hefei, Anhui, China; ^3^Division of Life Sciences and Medicine, School of Life Sciences, University of Science and Technology of China, Hefei, Anhui, China; ^4^State Key Laboratory of Cognitive Neuroscience and Learning and IDG/McGovern Institute for Brain Research, Beijing Normal University, Beijing, China; ^5^SILC Business School, Shanghai University, Shanghai, China; ^6^Application Technology Center of Physical Therapy to Brain Disorders, Institute of Advanced Technology, University of Science and Technology of China, Hefei, China; ^7^Biomedical Sciences and Health Laboratory of Anhui Province, University of Science and Technology of China, Hefei, China

**Keywords:** artificial intelligence, brain, brain-inspired intelligence, neurobiological explainability, AI evaluation, artificial neural network

## Abstract

Fueled by the development of neuroscience and artificial intelligence (AI), recent advances in the brain-inspired AI have manifested a tipping-point in the collaboration of the two fields. AI began with the inspiration of neuroscience, but has evolved to achieve a remarkable performance with little dependence upon neuroscience. However, in a recent collaboration, research into neurobiological explainability of AI models found that these highly accurate models may resemble the neurobiological representation of the same computational processes in the brain, although these models have been developed in the absence of such neuroscientific references. In this perspective, we review the cooperation and separation between neuroscience and AI, and emphasize on the current advance, that is, a new cooperation, the neurobiological explainability of AI. Under the intertwined development of the two fields, we propose a practical framework to evaluate the brain-likeness of AI models, paving the way for their further improvements.

## Introduction

Artificial intelligence (AI) starts with the notion of creating Turing-powerful intelligent systems (Turing, 1936). He claimed that his desire was to build a machine to “imitate a brain” and also to “mimic the behavior of the human,” which means the likeness to both the brain and the behavior is requisite to realize such intelligent systems. For this to happen, pioneers in the field (Rosenblatt, 1958; Fukushima and Nixon, 1980; Bi and Poo, 1998; Masquelier and Thorpe, 2007) have drawn inspiration from the neurobiological representation to develop AI models. However, early models or algorithms strictly mimicking the neural processes in the brain have constantly failed to deliver satisfactory performances, such as the perceptron (Rosenblatt, 1958), Hebbian learning rules (Kempter et al., 1999), and Sigmoid (Han and Moraga, 1995). Gradually, computer scientists have strayed away from neuroscience and turned to engineering and mathematical solutions to design “outcome-driven” models. These models achieved remarkable performance in many aspects, including but not limited to object recognition (Riesenhuber and Poggio, 2000), speech and music recognition (Kell et al., 2018; Sutskever et al., 2019), and motor movement (Todorov, 2000).

Nonetheless, comparison between AI and the brain has never stopped. Once optimized performance is achieved, researchers (Yamins et al., 2014; Güçlü and van Gerven, 2015; Eickenberg et al., 2017; Zhuang et al., 2021) begin to search for the neurobiological explainability of these advanced models, that is, the similarity of the neurobiological representation of the same computational processes between AI models and the brain. The authors wish that through unraveling the neurobiological explainability of AI models, one could achieve a better understanding of the brain and thus promote the development of neuroscience (Lindsay, 2021). Interestingly, in return, the evaluation of resemblance between current AI models and the brain may also shed lights on how far away these models are to the Turing-powerful (i.e., brain-like) intelligent systems.

**FIGURE 1:**
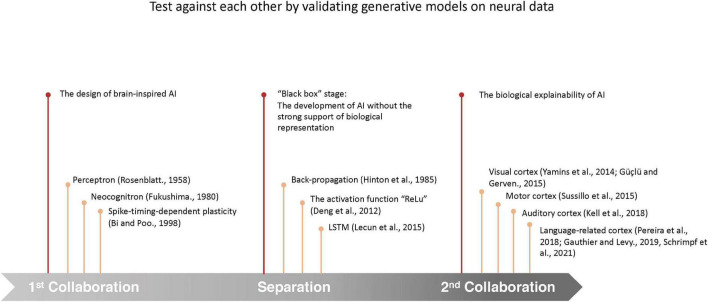
Timeline of the development of the interaction between neuroscience and AI. During the development, the interaction between neuroscience and AI has experienced 3 stages: (1) neuroscience guided the design of AI models; (2) implementations of engineering and mathematical tools instead of neurobiological principles in the development of AI models have led to substantial improvement in model performances, but the complex algorithms and huge parametric space behind the high performance pose a dilemma for the explanation of the underlying specific decision-making of the models, which are also called “black box” models (Arrieta et al., 2020); (3) by comparing the predicted neurobiological representation by modern AI models to the real neural processes in the brain, neurobiological explainability are provided to AI models.

During the three stages (Figure 1) of AI development, the role of neuroscience has experienced a shift from the “guide,” who provides guiding principles to the design of AI models, to the “judge,” who provide references for the evaluation of AI models. In this review, we will look back to the mutual development of AI and neuroscience, and propose a framework to evaluate the brain-likeness of AI models that can serve AI development in multiple ways.

## The collaboration and separation of artificial intelligence and neuroscience

Brain is the most complex and efficient non-artificial intelligent system known to humans. Throughout history, the promise of creating machine intelligence with brain-like ability has been a motivation of innovation (Roy et al., 2019). One way to realize such intelligence is to scrutinize the organization principles of brain’s structures and functions and thus seek inspiration for the design of AI. Hassabis et al. (2017) stated that if a new facet of neurobiological representation were found, it would be considered as an excellent candidate for incorporation into AI. Over the years, AI models have been rapidly developed by drawing inspiration from the brain neural networks, whereas algorithms, architectures, and functions of models have benefited greatly from mimicking such neurobiological representations (e.g., neuro-synaptic framework and hierarchical structure).

In the initial collaboration between AI and neuroscience, the direct inspiration from neuroscience accelerated the start-up of AI. The earliest application was the perceptron (Rosenblatt, 1958), a simple abstract of neurons, mimicking the simple neuronal activity in visual cortex, such as the weights of synapses, the biases of the thresholds, and the activation function of the neural cells. Years later, inspired by Hubel and Wiesel’s (1962) study in the visual cortex, Fukushima and Nixon (1980) proposed an advanced model, Neocognitron, the precursor of the modern convolutional neural networks (CNN), which mimicked the organizations of neural cells in the visual cortex. Apart from the inspiration of how neurons activate, researchers also designed some brain-corresponding models (e.g., topographic maps) inspired by how brain is organized. For example, Burak and Fiete (2009) modeled the network topology of the rats entorhinal cortex to form the neural substrate for dead-reckoning.

Although AI is profoundly inspired by the neurobiological representation of the brain, surprisingly, these brain-mimicking models have never achieved a satisfactory performance, likely due to their over-simplification of the real neural system. For instance, Hebbian learning, a neurobiologically schemed method, fail to produce models with adequate performance as it does not take into consideration of the synapse’s downstream effect on the network output (Lillicrap et al., 2020). Gradually, researchers (Rumelhart et al., 1986; Hinton et al., 2012; Lecun et al., 2015) started to turn to engineering and mathematical solutions to maximize model performance regardless of its underlying neurobiological relevance. In these works, the authors replaced the former neurobiological schemed methods with back-propagation, an algorithm without a prior neurobiological relevance, and solved the low-efficiency problem of synaptic modification (Lillicrap et al., 2020). Moreover, replacing the former neurobiologically inspired Sigmoid function (Han and Moraga, 1995) with the activation function ReLu (Deng et al., 2010) has been demonstrated to substantially improve the performance of deep neural networks (DNNs) since Krizhevsky et al. (2012). Given such superior performances, are these models operated in anyway similar to the most efficient system we ever know, the brain, despite they are not strictly structured to follow any neurobiological principles?

## Neurobiological explainability of artificial intelligence

Despite of the turning of design principles from mimicking neurobiological representation of the brain to optimizing performance with tools from engineering and mathematics, AI and neuroscience have never really grown apart. With the rapid development of AI, researchers (Yamins et al., 2014; Güçlü and van Gerven, 2015; Eickenberg et al., 2017; Zhuang et al., 2021) believe that these advanced models are capable to promote the development of neuroscience in return. In specific, they advocate for seeking for the neurobiological explanations for AI models as an alternative way to better understand the organization principles of the brain.

Early studies exploring the neurobiological explainability of AI models have mainly focused on visual recognition. Yamins et al. (2014) first examined the similarity between real brain activities and predicted activations from CNN model. The authors trained CNN model to match human performances on various visual recognition tasks. The results showed that the third and the fourth (top) layer of the model could effectively predict the inferior temporal activity recorded with functional MRI during image recognition. Other findings (Cadieu et al., 2014; Khaligh-Razavi and Kriegeskorte, 2014; Güçlü and van Gerven, 2015) also confirmed that deep neural network (DNN) models trained for visual recognition have remarkable predictability for the neural responses in the human visual system as well. Moreover, Cichy et al. (2016) found that the predicted brain activities by DNN trained for object categorization are highly resemble to the brain activations recorded *via* both fMRI and MEG during the same cognitive process, not only in the physical space domain (i.e., matching the hierarchical topography in the human ventral and dorsal visual streams), but also in the temporal domain (i.e., matching the time course over visual processing).

In addition to visual recognition, models designed for other utilities also showed similar predicted neurobiological representations with the real activities in the corresponding neural systems. A recent heavily focused area is the neurobiological explainability of AI models for language processing, including syntax processing (Gauthier and Levy, 2019), semantic processing (Pereira et al., 2016; de Heer et al., 2017), and comprehension (Schrimpf et al., 2021). Adding to these evidence, highly corresponded mappings between predicted (by the AI) and recorded (in the brain) neurobiological representations have also been found in other cognitive systems, such as the auditory system (Kell et al., 2018), the motor system (Sussillo et al., 2015), and even the hippocampal formation (Whittington et al., 2021).

## Quantify the progress toward Turing-powerful intelligence

Such demonstration of neurobiological explainability of AI models has opened the door for new contributions from neuroscience, to provide alternative tools to quantitatively evaluate the progress we made toward the Turing-powerful intelligent systems. Normally, evaluation to the distance to such intelligent systems concentrates at the behavioral level, where the performance of models would be evaluated, such as model-model comparison and model-to-human behavior comparison. However, the neurobiological explainability gives us cues to evaluate the brain-likeness of the models, which mainly focused on whether they can solve the same problems as the brain. Evaluation at the both behavioral and neurobiological gives us a more comprehensive insight to evaluate the distance to the Turing-powerful intelligent systems. Besides, the improvements of the algorithm can also indicate the advancement toward the Turing-powerful intelligent systems. To further elaborate on this new role of neuroscience in AI development, here, we capitalize on the Marr’s (1982) widely recognized computational framework, and discuss such applications in three levels.

### Evaluation at the computational level

In Marr’s theory, the first level, computational level, concerns the problems that models can solve. The evaluation of the performance of the models can be categorized into two ways: model-to-model comparison and model-to-human behavior comparison. The model-to-model comparison literally compares performances of different models for the same task. For instance, Xu et al. (2021) compared supervised models to unsupervised models and found that the latter trained with 10 min of labeled data, could rival the best supervised model trained with 960 h of labeled data. The model-to-human behavior comparison contrasts AI performance to human performance during the same task. For instance, Rajalingham et al. (2018) compared the ANNs’ (Artificial neural networks) accuracy in the visual categorization task with the behavioral results from 1,477 primates (1,472 humans and 5 monkeys), and evaluated that the models could not achieve the human-like behavioral performance.

### Evaluation at the algorithmic level

The second level of Marr’s theory, algorithmic level, concerns the processes that models go through. During the exploration into the neurobiological explainability of models, the training methods for models displayed a positive shift, implying that models turned out to be more intelligent. First, the training methods for models in the earlier studies aimed to map the computational models into the corresponding brain activity (Mitchell et al., 2008) when receiving the same stimuli, or to use the brain responses to constrain the models (Cadieu et al., 2014). And then the artificial neural response generated from models and the unlearned brain data were compared. However, in more recent studies, researchers (Yamins et al., 2014; Kell et al., 2018; Schrimpf et al., 2021) start to train AI models with only behavioral (e.g., objects and their labels) but not any neuroimaging data. Interestingly, while the models were not optimized to fit brain signals in the first places, they can still predict the brain responses during the same cognitive process proficiently. These findings suggest that the computational processes of these models can be brain-like enough to generate neurobiological representation without explicit training.

Furthermore, the shift of paradigm from supervised to unsupervised models during the prediction of neurobiological representation can also be seen as a step-forward toward brain-like intelligence, since the latter is considered to be more similar to human learning pattern which is constantly exposed to unlabeled environments (Mitchell, 2004), which could even automatically learn the human bias from image classification (Steed and Caliskan, 2021). In earlier studies, models used to predict neurobiological representation were mostly supervised models (Cadieu et al., 2014; Yamins et al., 2014; Güçlü and van Gerven, 2015). A study even suggested that unsupervised models could not predict the brain responses (Khaligh-Razavi and Kriegeskorte, 2014). However, with the improvement of unsupervised models during the decade (Xu et al., 2021), recent studies have found evidence that unsupervised models could successfully predict the neural response as well. For instance, Zhuang et al. (2021) found that the unsupervised models achieved a high prediction accuracy in the primate ventral stream that equaled and even surpassed the performance of the best supervised model. Thus, the recent success of unsupervised models in predicting brain representation suggests that AI models have made a giant step forward on the human-like path.

### Evaluation at the implementation/physical level

The last level of Marr’s theory, implementation/physical level, concerns the brain-likeness of the models.

First, instantiation (i.e., the neural representation of models) of the brain-inspired model would be an explicit and effective measure for judging success and spurring the progress to the Turing-powerful intelligence. It is an explicit measure since the layers in the models almost correspond to the hierarchical structure of the brain (Kell et al., 2018), where we can directly compared the detailed performance in each layer with the corresponding responses in the brain. If a model could highly predict the response in the brain, we consider that its corresponding parameters/weight would be vital to achieve the Turing-powerful intelligent systems (Hassabis et al., 2017). It is also an effective measure to drive models toward the goal. For example, Yamins et al. (2014) found that the top layer in the model could better predict the activity in IT cortex while other layers did not achieve the satisfactory performance. In this case, we may allocate more energy to optimize the layers that cannot successfully explain the corresponding neurobiological representation, which determines the most productive way to allocate resources.

Second, the evaluation of models from the perspective of neuroscience further supports the validation of behavioral results. Many studies have indicated that the more brain-like the model is, the better performance the model has in the task. For instance, Yamins et al. (2014) indicated that when a model highly predicts the IT (Inferior temporal) cortex, the better performance it would have in the object recognition task. And the same results were also found in another study (Khaligh-Razavi and Kriegeskorte, 2014). They compared 37 models with the human’s and monkeys’ cortex, respectively, showing that the models with more relevant correspondences with the neural representation in IT cortex have better performance in object recognition. Further, in the language model, Schrimpf et al. (2021) compared the predictability for neural response between 43 diverse language models, where they found models with high next-word predictive ability, like GPT models, have a better performance in predicting brain signals in language comprehension. Even they compared the ability of the next-word prediction of these models in another dataset, the neural predictivity still significantly correlated with the behavioral results. The parallel but highly correlated results provide us an opportunity to evaluate and further modify models from another perspective, neuroscience. Combining with the first point, it gives us a sight that we may modify the models more brain-like in order to achieve better performance.

Third, the evaluation at the behavioral level may not comprehensively explain the brain-like intelligence, as the way to process information differs in the brain and behavior (Bechara et al., 1997; Soon et al., 2008). Researchers claimed that the unconscious biases observed in the brain guided behavior before the conscious knowledge did, which means the brain signal might capture the subtle differences that were obscure at the behavioral level. Thus, the evaluation at the neurobiological level may evaluate the distance to the Turing-powerful intelligence more accurately compared to the behavioral evaluation.

## Implications for the improvement of AI models

Thus far, we have reviewed the collaboration and separation between neuroscience and AI, and highlighted the significance of the current collaboration. More importantly, we propose the importance of evaluating models from the perspective of neuroscience. The evaluation tells us the closeness between the current models and the brain, which is critical to optimize models in achieving the Turing-powerful level.

**FIGURE 2:**
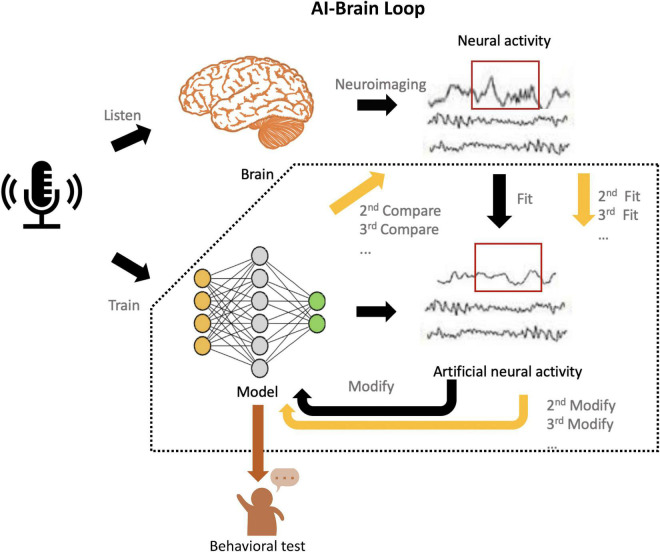
Schematic of the AI-Brain loop. First, present the same task (e.g., audio recognition) to human subjects and AI models, which are subsequently trained for this task. Second, record the neural activities in the brain by neuroimaging techniques (e.g., fMRI., EEG., MEG., ECoG), and predict neural responses with these trained AI models. Third, compare the recorded neural activity and the artificial neural activity generated by models. Fourth, use the artificial neural activity to fit the neural activity by modifying the corresponding layers or parameters. Fifth, implement the behavioral evaluation of model and see whether the performance achieve the human-like level. If not, implement the continuous fit until it achieves the both human-like and brain-like level.

To move forward, here, we present an AI-brain loop framework in which we implement the explicit evaluation from neuroscience and accurate modification in each layer and even parameters to the models (Figure 2), inspired by the human-in-the-loop (Li et al., 2014) and inception loop (Walker et al., 2019).

In this framework, we propose that the AI models trained for specific behavioral task can use neural recordings during the same task as neurobiological reference. Comparisons between recorded and model-predicted neural responses can be used to tune the parameter space of the AI models, and more realistic neurobiological representation of the models can be achieved during the process of minimizing the differences between the two. Lastly, performance of the modified models at behavioral level will be used to verify that whether models with higher brain-likeness level, but also function at the human-like level. Then we also test the modified models at the behavioral level and see whether the performance improves. Such clues of the modification are fundamental to achieve the Turing-powerful intelligent system since it echoes Turing’s claim (Turing, 1936) that models are qualified in not only “mimicking the behavior of the human,” but also “imitating the brain.” The call for Turing-powerful intelligent system asks to look beyond performance optimization, but to focus more on how to achieve higher brain resemblance in future AI models. We believe that re-introducing neuroscience back into AI development through this neurobiological explainability provides a promising opportunity to outbreaking the “black-box” dilemma suffered by most of modern AI models. By “jumping out of the box” and developing more brain-like AI through such AI-brain comparisons, we may eventually leap forward to such ultimate goal.

## Author contributions

YC, ZW, XH, and XZ contributed to the conception of the study. YC and ZW wrote the first draft of the manuscript. YC, ZW, and XH wrote sections of the manuscript. YC, ZW, HG, HL, LG, XH, and XZ contributed to revise the manuscript and customized the tables. All authors contributed to manuscript revision, read, and approved the submitted final versions.
